# Neurofilament, but not Alzheimer disease biomarkers in the acute phase correlate with cognitive performance after cardiac arrest

**DOI:** 10.1016/j.resplu.2025.101025

**Published:** 2025-07-08

**Authors:** Johannes Lorentzson, Gisela Lilja, Erik Blennow Nordström, Kaj Blennow, Henrik Zetterberg, Christian Hassager, Matt P. Wise, Andrea L. Benedet, Tommaso Pellis, Hans Friberg, Nicholas Ashton, Marion Moseby Knappe

**Affiliations:** a Department of Neurology, Skane University Hospital, Lund, Sweden; b Department of Clinical Sciences Lund, Lund University, Lund, Sweden; c Department of Rehabilitation Medicine, Skane University Hospital, Lund, Sweden; d Clinical Neurochemistry Laboratory, Sahlgrenska University Hospital, Mölndal, Sweden; e Institute of Neuroscience and Physiology, University of Gothenburg, Mölndal, Sweden; f Paris Brain Institute, ICM, Pitié-Salpêtrière Hospital, Sorbonne University, Paris, France; g Neurodegenerative Disorder Research Center, Division of Life Sciences and Medicine, and Department of Neurology, Institute on Aging and Brain Disorders, University of Science and Technology of China and First Affiliated Hospital of USTC, Hefei, PR China; h Department of Psychiatry and Neurochemistry, Institute of Neuroscience and Physiology, the Sahlgrenska Academy at the University of Gothenburg, Mölndal, Sweden; i Department of Neurodegenerative Disease, UCL Institute of Neurology, Queen Square, London, United Kingdom; j UK Dementia Research Institute at UCL, London, United Kingdom; k Hong Kong Center for Neurodegenerative Diseases, Clear Water Bay, Hong Kong, China; l Wisconsin Alzheimer’s Disease Research Center, University of Wisconsin School of Medicine and Public Health, University of Wisconsin. Madison, Madison, WI, USA; m Department of Cardiology, Rigshospitalet, Copenhagen, Denmark; n Department of Clinical Medicine, University of Copenhagen, Copenhagen, Denmark; o Adult Critical Care, University Hospital of Wales, Heath Park, Cardiff, United Kingdom; p Department of Anaesthesiology and Intensive Care, Pordenone Hospital, Azienda Sanitaria Friuli Occidentale, Italy; q Department of Anaesthesiology and Intensive Care, Skane University Hospital, Malmö, Sweden; r Banner Alzheimer’s Institute and University of Arizona, Phoenix, AZ, USA; s Banner Sun Health Research Institute, Sun City, AZ 85351, USA

**Keywords:** OHCA, Neuroprognostication, Amyloid beta, Tau, Neurofilament light, Cognitive impairment

## Abstract

**Background:**

Biomarkers serve as a quantitative measure of brain injury and may predict cognitive outcome after cardiac arrest. This study investigates the association and predictive accuracy of acute changes in Alzheimer disease-associated biomarkers to cognitive outcome in cardiac arrest survivors.

**Methods:**

Retrospective study of the Target Temperature Management after Out-of-Hospital cardiac arrest trial. Serum from adult cardiac arrest survivors was sampled prospectively at 24, 48, and 72 h post-arrest and analyzed for peak-levels of Alzheimer disease markers (p-tau^181^, total tau, amyloid β [Aβ40 and Aβ42]), and the neurodegenerative biomarker neurofilament light (NfL). Cognitive outcome was evaluated blinded from biomarker results using four performance-based assessments at 6 months post-arrest. Spearman correlations were calculated. Area Under the Receiver Operating Characteristics curves (AUC) were calculated for biomarkers discriminatory ability for binary results of cognitive performance.

**Results:**

206/342 (60 %) survivors from participating sites were included. Median was age 62 (IQR 53–69), 86 % male, 15 (7 %) had Mini-Mental State Examination (MMSE) scores < 24. Alzheimer disease biomarkers exhibited at best small correlations to cognitive outcomes (rho = −0.22 to 0.18). The correlation between outcome instruments and NfL was rho = −0.32 to −0.20 (*p* < 0.01). Discriminatory ability of cognitive impairment for acute changes in Alzheimer disease biomarkers was AUC 0.44–0.68 (95 % CI 0.29–0.82), and AUC 0.66–0.86 (95 % CI 0.59–0.95) for NfL.

**Conclusion:**

In contrast to tau- and amyloid-related biomarkers, NfL could be more useful for predicting cognitive function in cardiac arrest survivors. Low participation by survivors with severe brain injury may have influenced results.

## Introduction

Cardiac arrest survivors with an overall good functional outcome commonly report cognitive impairments, mainly observed as deficits in attention, memory or executive function affecting participation in everyday life and society, and the ability to return to work.^1^, ^2^ Guidelines recommend cognitive screening within three months after hospital discharge to optimize support.^3^ There is little evidence on how to identify individuals who may benefit from cognitive rehabilitation during intensive care.

Blood biomarkers serve as quantitative measures of brain injury and could potentially predict cognitive impairment.^3^, ^4^ Biomarkers phosphorylated tau at threonine 181 (p-tau^181^), total tau (t-tau), amyloid-beta peptides 40 (Aβ40) and 42 (Aβ42) demonstrate Alzheimer disease related changes and often correlate with cognitive decline, these are hereafter summarized as “Alzheimer disease biomarkers”.^5^

Neurofilament light (NfL), t-tau and p-tau^181^ in the acute phase following cardiac arrest were found predictive of poor functional outcome by the modified Rankin Scale.^6^, ^7^, ^8^ In the same analysis, Aβ40 and Aβ42 demonstrated increasing serum levels 48 and 72 h post-arrest among patients with good functional outcome, but their significance for cognitive performance remains unclear. NfL previously demonstrated small correlations with cognitive outcome measured by the Mini-Mental State Examination (MMSE).^9^ To our knowledge, the correlation between levels of Alzheimer disease biomarkers with long-term cognitive impairment after cardiac arrest has not been examined.

The aim of this study was to investigate the association and discrimination ability of acute changes in Alzheimer disease-associated blood biomarkers to cognitive impairment in cardiac arrest survivors.

## Methods

### Study cohort and design

This was a retrospective study of the international, multicenter Target Temperature Management after out-of-hospital cardiac arrest (TTM) trial including patients ≥ 18 years with a presumed cardiac origin of arrest.^10^, ^11^ Serum samples were prospectively collected at 24, 48 and 72 h post-arrest and stored in a biobank as described.^12^ At 6 months, all survivors from 17 participating European sites were invited to a face-to-face follow-up in the site language(s) including screening of cognitive outcome by assessors blinded to biomarker levels.^2^, ^10^, ^11^ The trial protocols were approved by ethics boards in all participating countries. Written informed consent was obtained before follow-up assessments.

### Biochemical analysis

As previously published, p-tau^181^, t-tau and NfL were measured on a Simoa HD-1 Analyzer (Quanterix) using Human Total Tau kit and Homebrew kit, respectively.^6^, ^7^, ^13^ Aβ40, Aβ42 and t-tau serum levels were measured with Neurology 3-Plex A Advantage Kit on the Simoa HD-1 Analyzer (Quanterix).^8^

### Cognitive outcome assessment

1) The MMSE was used for cognitive screening.^14^ Its score ranges from 0 to 30, a cut-off < 24 often used in dementia research, was applied to indicate a high likelihood of cognitive impairment.^15^
2) Rivermead Behavioural Memory Test (RBMT) assesses everyday memory. The score ranges from 0 to 24 points, with RBMT < 17 set as the cut-off to identify moderately to severely impaired memory function.^16^.
3) Symbol Digit Modalities Test (SDMT) assesses attention and processing speed. The cut-off was set to ≤ −1.5 z-scores in age and educational level-adjusted populations, indicating possible cerebral injury.^17^.
4) The score of the Frontal Assessment Battery (FAB) ranges from 0 to 18, higher scores indicate better performance.^18^ We used a cut-off score < 12 since previous studies suggested this cut-off to distinguish between frontotemporal dementia and Alzheimer disease.^19^

### Statistical analysis

The association between peak-biomarker levels (the highest concentration available at 24, 48 and 72 h), or fold change (change in concentration between two timepoints) and the outcome of cognitive assessments were evaluated with Spearman’s Rank Correlation. The absolute values are reported as followed: < 0.10 = trivial, 0.10–0.29 = small, 0.30–0.49 = moderate, and ≥ 0.50 = large.^20^

Receiver Operating Characteristics (ROC) curves from univariate logistic regression analysis are presented for peak-biomarker levels for discrimination ability of the four cognitive assessments according to the binary cutoffs specified above. Scores from included participants with missing data on the assessments were imputed as performance under the binary cut-offs in the logistic regression analysis.

Two-tailed *p* < 0.05 was considered statistically significant. Statistical analyses were performed using R version 4.2.2 (The R Foundation for Statistical Computing).

## Results

**Fig. 1:**
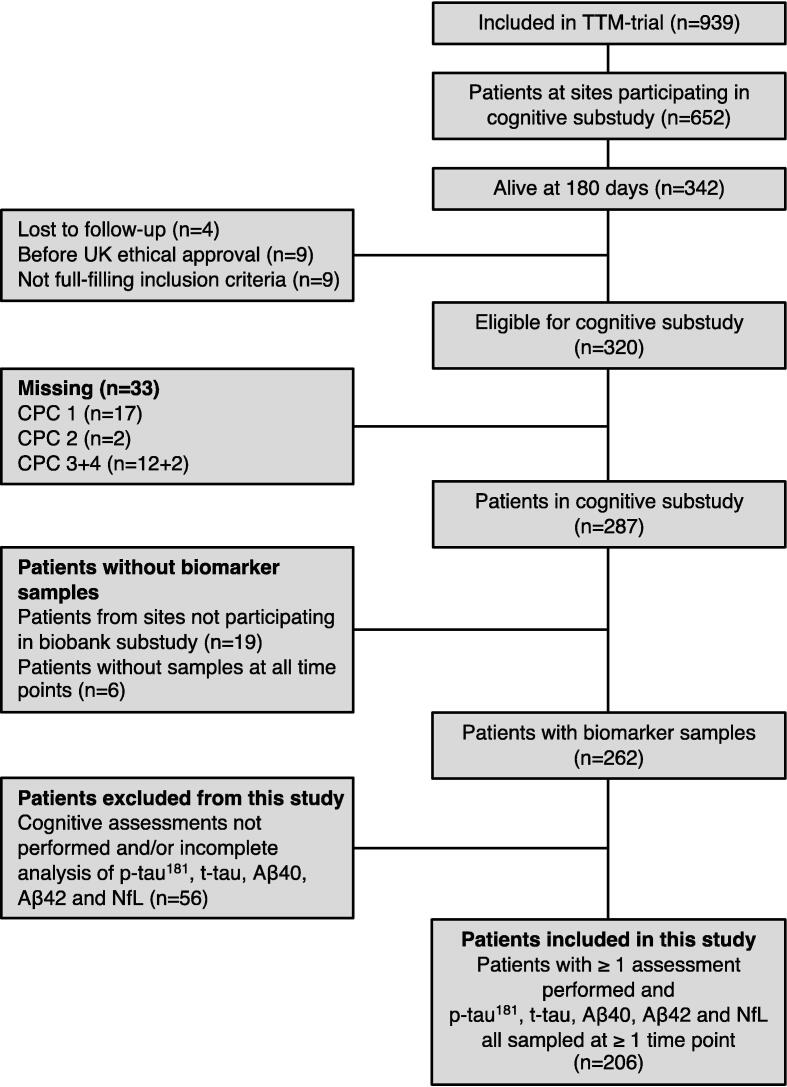
**Flowchart of patient inclusion.** Abbreviations: TTM trial – Targeted Temperature Management at 33 °C vs 36 °C after out-of-hospital cardiac arrest trial, CPC – Cerebral Performance Category scale, p-tau^181^ – phosphorylated tau at threonine 181, t-tau – total tau, Aβ40 – amyloid-beta 40, Aβ42 – amyloid-beta 42, NfL – neurofilament light chain. Biomarkers at 24, 48 and 72 h were available for 97–98 %, 93–95 % and 88–91 % of participants, respectively.

We included 206/342 (60 %) of survivors from participating sites who had biomarker results and completed ≥ 1 cognitive assessment (Fig. 1). Biomarker levels, performance of the cognitive assessments including indicated cognitive impairment according to applied cut-offs, and clinical data of included and excluded patients are presented in Table 1. The MMSE was the only instrument administrated to all included participants, while 193/206 (94 %) performed all four cognitive assessments. Patients completing all cognitive assessments generally performed better than participants who performed ≤ 3 assessments. Peak-NfL levels were in median 61 pg/mL (IQR: 32–124) in participants performing all four assessments, compared to median 241 pg/mL (IQR: 88–1131) in patients performing ≤ 3 assessments.

**Table 1:** Patient characteristics of study population, biomarker levels, and performance of the cognitive screening assessments.

	**Included (*n* = 206)**			**Excluded (*n* = 56)**
	**All** (*n* = 206)	≤**3 assessments performed** (*n* = 13)	**All 4 assessments performed** (*n* = 193)	
Age (*years*)	62 (53–69)	66 (64–78)	61 (52–68)	63 (58–69)
Male	178 (86.4)	10 (76.9)	168 (87.0)	48 (85.7)
< 12 years of education	117 (57.1)	11 (84.6)	106 (54.9)	30 (54.5)

**Medical history**				
Previous acute MI	38 (18.4)	6 (46.2)	32 (16.6)	5 (8.9)
Ischemic heart disease	50 (24.3)	7 (53.8)	43 (22.3)	10 (17.9)
Arterial hypertension	81 (39.3)	4 (30.8)	77 (39.9)	19 (33.9)
Diabetes mellitus	28 (13.6)	3 (23.1)	25 (13.0)	6 (10.7)

**Cardiac arrest characteristics**				
Minutes to ROSC	20 (15–30)	25 (11–37)	20 (15–30)	21 (15–27)
Bystander CPR	164 (79.6)	9 (69.2)	155 (80.3)	45 (80.4)
Shockable rhythm	191 (94.6)	11 (84.6)	180 (95.2)	52 (98.1)

**Peak-biomarker levels** (*pg/mL*)				
p-tau^181^	7.4 (5.7–10.4)	7.1 (5.9–16.6)	7.4 (5.7–10.3)	8.1 (6.9–13.4)
t-tau	2.7 (1.7–5.7)	4.3 (1.7–13.0)	2.7 (1.8–5.5)	3.2 (1.8–5.6)
Aβ40	177 (121–260)	205 (156–308)	174 (119–258)	min 43.8, max 291
Aβ42	9.0 (6.3–12.4)	7.5 (6.4–11.7)	9.1 (6.3–12.4)	min 1.2, max 14.3
NfL	62.2 (32.7–141.7)	240.9 (87.5–1131.0)	60.6 (32.3–124.4)	108.7 (24.5–201.7)

**Occupational status** (*pre-arrest / 6 months*)				
Working full-time	90 (43.7) / 42 (20.4)	3 (23.1) / 0 (0.0)	87 (45.1) / 42 (21.8)	23 (41.8) / 11 (20.0)
Working part-time	15 (7.3) / 29 (14.1)	0 (0.0) / 0 (0.0)	15 (7.8) / 29 (15.0)	4 (7.3) / 8 (14.5)
Unemployed	7 (3.4) / 8 (3.9)	0 (0.0) / 0 (0.0)	7 (3.6) / 8 (4.1)	1 (1.8) / 1 (1.8)
Retired	87 (42.2) / 92 (44.7)	9 (69.2) / 10 (76.9)	78 (40.4) / 82 (42.5)	25 (45.5) / 27 (49.1)
On sick leave	6 (2.9) / 34 (16.5)	1 (7.7) / 3 (23.1)	5 (2.6) / 31 (16.1)	2 (3.6) / 8 (14.5)
Other	1 (0.5) / 1 (0.5)	0 (0.0) / 0 (0.0)	1 (0.5) / 1 (0.5)	0 (0.0) / 0 (0.0)

**modified Rankin Scale at follow-up**				
Binary outcome				
Good (mRS 0–3)	196 (95.1)	6 (46.2)	190 (98.4)	49 (87.5)
Poor (mRS 4–6)	10 (4.9)	7 (53.8)	3 (1.6)	7 (12.5)
mRS score at follow-up				
0 (no symptoms)	76 (36.9)	0 (0.0)	76 (39.4)	18 (32.1)
1 (slight disability)	73 (35.4)	1 (7.7)	72 (37.3)	19 (33.9)
2	36 (17.5)	4 (30.8)	32 (16.6)	8 (14.3)
3 (moderate disability)	11 (5.3)	1 (7.7)	10 (5.2)	4 (7.1)
4	9 (4.4)	6 (46.2)	3 (1.6)	1 (1.8)
5 (severe disability)	1 (0.5)	1 (7.7)	0 (0.0)	6 (10.7)

**Test performance at follow-up**				
MMSE	29.0 (27.0–30.0)	22.0 (14.8–29.2)	29.0 (27.0–30.0)	28.0 (25.0–30.0)
RBMT	21.0 (18.0–23.0)	5.0 (2.5–14.0)	21.0 (18.0–23.0)	20.5 (16.8–23.2)
SDMT	−1.0 (−2.0–−0.4)	−1.6 (−2.8–−1.6)	−1.0 (−2.0–−0.4)	−1.2 (−1.8–−0.4)
FAB	17.0 (15.0–18.0)	10.0 (4.0–16.5)	17.0 (15.0–18.0)	17.0 (13.5–18.0)

**Patients with test performance below cut-off**				
MMSE < 24	15 (7.3)	6 (50.0)	9 (4.7)	11 (21.2)
RBMT < 17	33 (16.8)	2 (66.7)	31 (16.1)	13 (25.0)
SDMT ≤ −1.5 z-scores	76 (38.8)	3 (100.0)	73 (37.8)	18 (36.0)
FAB < 12	15 (7.4)	6 (54.5)	9 (4.7)	8 (15.7)

Continuous variables are presented as median (IQR) and categorical variables as n (%). Occupational status is presented for pre-arrest and 6 months after cardiac arrest. Abbreviations: MI – myocardial infarction, ROSC – return of spontaneous circulation, CPR – cardiopulmonary resuscitation, p-tau^181^ – phosphorylated tau at threonine 181, t-tau – total tau, Aβ40 – amyloid-beta 40, Aβ42 – amyloid-beta 42, NfL – neurofilament light chain, mRS – modified Rankin Scale, MMSE – Mini-Mental State Examination, RBMT – Rivermead Behavioural Memory Test, SDMT – Symbol Digit Modalities Test, FAB – Frontal Assessment Battery.

### Association between biomarkers and cognitive outcome instruments

The correlation between peak-levels of Alzheimer disease biomarkers and the cognitive outcome assessments was rho = −0.22 to 0.18, while the correlations for NfL was rho = −0.32 to −0.20 (*p* < 0.01), Table 2. The correlation between fold change in biomarker levels was only statistically significant for t-tau and the RBMT (rho = −0.15, *p* < 0.05) and the FAB (rho = −0.21, *p* < 0.01), eTable 1-2.

**Table 2:** Spearman correlations for peak-biomarker levels and results on the four cognitive outcome instruments.

**Instruments**	**p-tau^181^**		**t-tau**		**Aβ40**		**Aβ42**		**NfL**	
	rho	Degree of correlation	rho	Degree of correlation	rho	Degree of correlation	rho	Degree of correlation	rho	Degree of correlation
MMSE	−0.16*	Small	−0.16*	Small	−0.07	Trivial	−0.01	Trivial	−0.32***	Moderate
RBMT	−0.04	Trivial	−0.11	Small	0.15*	Small	0.18*	Small	−0.20**	Small
SDMT	−0.12	Small	−0.09	Trivial	−0.15*	Small	−0.15*	Small	−0.29***	Small
FAB	−0.22**	Small	−0.09	Trivial	−0.01	Trivial	0.07	Trivial	−0.30***	Moderate

* *p* < 0.05; ** *p* < 0.01; *** *p* < 0.001; Abbreviations: p-tau^181^ – phosphorylated tau at threonine 181, t-tau – total tau, Aβ40 – amyloid-beta 40, Aβ42 – amyloid-beta 42, NfL – neurofilament light chain, MMSE – Mini-Mental State Examination, RBMT – Rivermead Behavioural Memory Test, SDMT – Symbol Digit Modalities Test, FAB – Frontal Assessment Battery.

### Biomarkers discrimination ability for binary cognitive performance

**Fig. 2:**
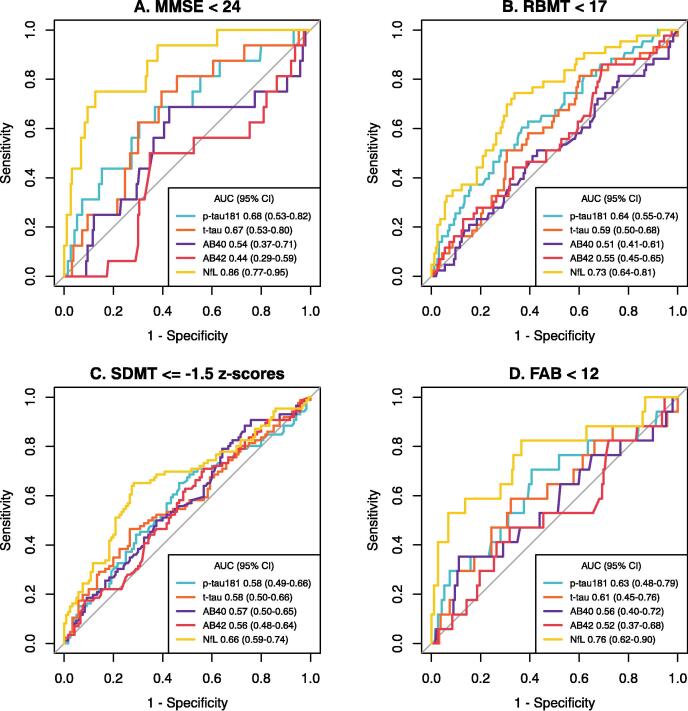
**A-D. Receiver Operating Characteristics curves for prediction of cognitive outcomes.** Performance of peak-serum levels of biomarkers to predict results on four cognitive outcome instruments at 6 months post-arrest presented as Area Under the Receiver Operating Characteristics curve (AUC) with 95 % confidence intervals (CI). Fig. 2A: MMSE – Mini-Mental State Examination dichotomized at cut-off < 24; Fig. 2B: RBMT – Rivermead Behavioural Memory Test dichotomized at cut-off < 17; Fig. 2C: SDMT – Symbol Digit Modalities Test dichotomized at cut-off ≤ −1.5 z-scores adjusted for patient age and educational level; Fig. 2D: FAB – Frontal Assessment Battery dichotomized at cut-off < 12. p-tau^181^ – phosphorylated tau at threonine 181, t-tau – total tau, AB40 – amyloid-beta 40, AB42 – amyloid-beta 42, NfL – neurofilament light.

The Alzheimer disease biomarkers discrimination ability for cognitive outcome is displayed in Fig. 2A-D.

NfL showed discrimination ability AUC 0.86 (95 % CI 0.77–0.95) by MMSE and discrimination ability AUC 0.73 (95 % CI 0.64–0.81) and 0.76 (95 % CI 0.62–0.90), for RBMT and FAB, respectively. None of the biomarkers demonstrated useful discrimination ability by patient age and educational level adjusted SDMT.

## Discussion

In contrast to the neurodegenerative marker NfL, Alzheimer disease biomarkers collected in the acute phase between 24 and 72 h after cardiac arrest did not demonstrate useful discrimination ability for cognitive function in this study.

Based on the previously high discrimination ability of p-tau^181^ and t-tau for functional outcome using the modified Rankin Scale after cardiac arrest, we had expected better correlations between Alzheimer disease biomarkers and cognitive assessments.^6^, ^8^ Another small study with 21 patients previously found elevated t-tau in cardiac arrest survivors with MMSE < 28 at 2 weeks post-arrest.^21^ In our study, the correlations between the four Alzheimer disease biomarkers and cognitive assessments were small. Fold changes of biomarker levels only significantly correlated with results of RMBT and FAB for t-tau, indicating that for t-tau, biomarker dynamics may be relevant.^22^

Even in absence of ischaemic injury, amyloid-beta peptides have low robustness to predict amyloid pathology typical for Alzheimer’s disease when measured in blood compared to cerebrospinal fluid.^23^ In the acute phase of hypoxic-ischemic brain injury, a disruption to the blood–brain barrier may further reduce the correlation of Alzheimer disease biomarkers and cognitive performance.^8^

Interestingly, the marker of axonal neurodegeneration, NfL, significantly correlated with all four cognitive outcome instruments, with higher correlations for MMSE and FAB. In our previous study with overlapping cohorts, NfL exhibited greater association to clinician-reported functional outcome than MMSE or patient- and observer-reported outcome.^9^ Our survivors who did not participate in all four assessments seemed to have higher levels of biomarkers and higher levels of cognitive impairment than those completing all assessments, and we thus included these patients with likely cognitive impairment in the ROC curves. We acknowledge that subgroup analyses are limited by the small number of patients with cognitive impairments. Further, the correlation between biomarkers and results on cognitive assessments were not as strong as with binary functional outcome, most likely resulting from high mortality of patients with the highest levels of brain injury markers.

The evaluation of discrimination abilities of biomarkers varies with the type of cognitive assessment and the cutoffs chosen. Many participants achieve scores in the upper limit of the MMSE scale and the sensitivity to identify milder cognitive impairment is questioned.^24^ The SDMT identified most cases with cognitive impairment in this study, yet none of the biomarkers examined demonstrated relevant discrimination ability by the chosen age- and educational level-adjusted cut-offs.^17^

Strengths of this study include the prospective sampling of biomarkers, blinded evaluation of cognitive assessments and the availability of five different biomarkers. Limitations include the selection of a subgroup of survivors with a presumed cardiac cause of arrest and favorable functional outcomes, as determined by the modified Rankin Scale. Only 7.3 % of participants had cognitive impairment, some of which had high biomarker levels and were unable to complete all administrated assessments. The pre-arrest cognitive performance and possible pre-existing neurodegenerative disease of participants were unknown. Regardless of the pathophysiological cause, patients may benefit from early identification of cognitive impairments. Access to rehabilitation is generally low after cardiac arrest, but we presume that some patients may have received cognitive rehabilitation.^25^ This was an exploratory study of acute biomarkers and their correlation with cognitive performance after cardiac arrest. We cannot exclude that the use of frozen samples may have negatively influenced our results.^26^ Biomarkers collected at the timepoint of cognitive assessments may better reflect cognitive performance than those collected in the acute phase as in the current study.

## Conclusion

In contrast to tau- and amyloid-related biomarkers, NfL could be more useful for predicting cognitive function in cardiac arrest survivors. Low participation by survivors with severe brain injury may have influenced results.

## Funding/support

The Target Temperature Management trial was funded by the Swedish Research Council, the Swedish Heart Lung Foundation, Arbetsmarknadens Försäkringsaktiebolag Insurance Foundation, the Skåne University Hospital Foundations, the Gyllenstierna-Krapperup Foundation, and governmental funding of clinical research within the Swedish National Health System, the County Council of Skåne; the Swedish Society of Medicine; the Koch Foundation; TrygFonden; European Clinical Research Infrastructures Network; Thelma Zoega Foundation; Stig and Ragna Gorthon Foundation; Thure Carlsson Foundation; Hans-Gabriel and Alice Trolle-Wachtmeister Foundation for Medical Research; Lions Research Fund Skåne; South Swedish Hospital Region Research Funds; the Swedish Brain Foundation; the Lundbeck Foundation; and the Torsten Söderberg foundation at the Royal Swedish Academy of Sciences.

HZ is a Wallenberg Scholar and a Distinguished Professor at the Swedish Research Council supported by grants from the Swedish Research Council (#2023-00356, #2022-01018 and #2019-02397), the European Union’s Horizon Europe research and innovation programme under grant agreement No 101053962, Swedish State Support for Clinical Research (#ALFGBG-71320), the Alzheimer Drug Discovery Foundation (ADDF), USA (#201809-2016862), the AD Strategic Fund and the Alzheimer's Association (#ADSF-21-831376-C, #ADSF-21-831381-C, #ADSF-21-831377-C, and #ADSF-24-1284328-C), the European Partnership on Metrology, co-financed from the European Union’s Horizon Europe Research and Innovation Programme and by the Participating States (NEuroBioStand, #22HLT07), the Bluefield Project, Cure Alzheimer’s Fund, the Olav Thon Foundation, the Erling-Persson Family Foundation, Familjen Rönströms Stiftelse, Stiftelsen för Gamla Tjänarinnor, Hjärnfonden, Sweden (#FO2022-0270), the European Union’s Horizon 2020 research and innovation programme under the Marie Skłodowska-Curie grant agreement No 860197 (MIRIADE), the European Union Joint Programme – Neurodegenerative Disease Research (JPND2021-00694), the National Institute for Health and Care Research University College London Hospitals Biomedical Research Centre, the UK Dementia Research Institute at UCL (UKDRI-1003), and an anonymous donor.

KB is supported by the Swedish Research Council (#2017-00915 and #2022-00732), the Swedish Alzheimer Foundation (#AF-930351, #AF-939721, #AF-968270, and #AF-994551), Hjärnfonden, Sweden (#FO2017-0243 and #ALZ2022-0006), the Swedish state under the agreement between the Swedish government and the County Councils, the ALF-agreement (#ALFGBG-715986 and #ALFGBG-965240), the European Union Joint Program for Neurodegenerative Disorders (JPND2019-466-236), the Alzheimer’s Association 2021 Zenith Award (ZEN-21-848495), the Alzheimer’s Association 2022-2025 Grant (SG-23-1038904 QC), La Fondation Recherche Alzheimer (FRA), Paris, France, the Kirsten and Freddy Johansen Foundation, Copenhagen, Denmark, and Familjen Rönströms Stiftelse, Stockholm, Sweden.

## Conflicts of interest

HZ has served at scientific advisory boards and/or as a consultant for Abbvie, Acumen, Alector, Alzinova, ALZpath, Amylyx, Annexon, Apellis, Artery Therapeutics, AZTherapies, Cognito Therapeutics, CogRx, Denali, Eisai, LabCorp, Merry Life, Nervgen, Novo Nordisk, Optoceutics, Passage Bio, Pinteon Therapeutics, Prothena, Quanterix, Red Abbey Labs, reMYND, Roche, Samumed, Siemens Healthineers, Triplet Therapeutics, and Wave, has given lectures sponsored by Alzecure, BioArctic, Biogen, Cellectricon, Fujirebio, Lilly, Novo Nordisk, Roche, and WebMD, and is a co-founder of Brain Biomarker Solutions in Gothenburg AB (BBS), which is a part of the GU Ventures Incubator Program (outside submitted work).

KB has served as a consultant and at advisory boards for Abbvie, AC Immune, ALZPath, AriBio, Beckman-Coulter, BioArctic, Biogen, Eisai, Lilly, Moleac Pte. Ltd, Neurimmune, Novartis, Ono Pharma, Prothena, Quanterix, Roche Diagnostics, Sanofi and Siemens Healthineers; has served at data monitoring committees for Julius Clinical and Novartis; has given lectures, produced educational materials and participated in educational programs for AC Immune, Biogen, Celdara Medical, Eisai and Roche Diagnostics; and is a co-founder of Brain Biomarker Solutions in Gothenburg AB (BBS), which is a part of the GU Ventures Incubator Program, outside the work presented in this paper.

MPW has served on advisory boards for DRW diagnostics and Clinical Expert NICE Advice Service.

TP reports fees as speaker from BD.

No other conflicts of interest were reported.

## CRediT authorship contribution statement

**Johannes Lorentzson:** Writing – review & editing, Writing – original draft, Visualization, Formal analysis, Data curation, Conceptualization. **Gisela Lilja:** Writing – review & editing, Writing – original draft, Supervision, Resources, Project administration, Methodology, Investigation, Funding acquisition, Data curation, Conceptualization. **Erik Blennow Nordström:** Writing – review & editing, Writing – original draft, Supervision, Data curation, Conceptualization. **Kaj Blennow:** Writing – review & editing, Resources, Methodology, Investigation, Funding acquisition, Data curation. **Henrik Zetterberg:** Writing – review & editing, Resources, Methodology, Investigation, Funding acquisition, Data curation. **Christian Hassager:** Writing – review & editing, Data curation. **Matt P. Wise:** Writing – review & editing, Data curation. **Andrea L. Benedet:** Writing – review & editing, Methodology, Investigation, Data curation. **Tommaso Pellis:** Writing – review & editing, Data curation. **Hans Friberg:** Writing – review & editing, Project administration, Methodology, Investigation, Funding acquisition, Data curation. **Nicholas Ashton:** Writing – review & editing, Resources, Methodology, Investigation, Funding acquisition, Data curation. **Marion Moseby Knappe:** Writing – review & editing, Writing – original draft, Supervision, Resources, Project administration, Methodology, Investigation, Funding acquisition, Data curation, Conceptualization.

## Declaration of competing interest

The authors declare that they have no known competing financial interests or personal relationships that could have appeared to influence the work reported in this paper.
