# Supplementary material for: Neurofilament, but not Alzheimer disease biomarkers in the acute phase correlate with cognitive performance after cardiac arrest
Source: Resusc Plus. 2025 Jul 8;25:101025. doi: 10.1016/j.resplu.2025.101025 (PMC12284371; doi:10.1016/j.resplu.2025.101025)
Supplement: Supplementary Data 1 [file mmc1.docx]

Supplementary appendix

Lorentzson J. et al. Neurofilament, but not Alzheimer disease biomarkers in the acute phase correlate with cognitive performance after cardiac arrest 

**eTable 1.** Spearman’s Rank Correlations for biomarker fold change between 48 and 24 hours post-arrest and cognitive instruments

**eTable 2.** Spearman’s Rank Correlations for biomarker fold change between 72 and 24 hours post-arrest and cognitive instruments

**eTable 1.** **Spearman’s Rank Correlations for biomarker fold change between 48 and 24 hours post-arrest and cognitive instruments**

| **Instruments** | **p-tau^181^** | | **Aβ40** | | **Aβ42** | | **NfL** | | **t-tau** | |
| --- | --- | --- | --- | --- | --- | --- | --- | --- | --- | --- |
|  | rho | Degree of correlation | rho | Degree of correlation | rho | Degree of correlation | rho | Degree of correlation | rho | Degree of correlation |
| MMSE | 0.05 | Trivial | -0.09 | Trivial | -0.10 | Small | -0.11 | Small | 0.01 | Trivial |
| RBMT | -0.09 | Trivial | -0.03 | Trivial | 0.00 | None | -0.11 | Small | -0.13 | Small |
| SDMT | 0.07 | Trivial | -0.09 | Trivial | -0.10 | Small | -0.07 | Trivial | -0.04 | Trivial |
| FAB | 0.04 | Trivial | 0.06 | Trivial | 0.02 | Trivial | -0.11 | Small | -0.11 | Small |
| * p < 0.05; ** p < 0.01; *** p < 0.001; Abbreviations: p-tau^181^ – phosphorylated tau at threonine 181, Aβ40 – Amyloid-β 40, Aβ42 – Amyloid-β 42, NfL – neurofilament light chain, t-tau – total tau, MMSE – Mini-Mental State Examination, RBMT – Rivermead Behavioural Memory Test, SDMT – Symbol Digit Modalities Test, FAB – Frontal Assessment Battery. | | | | | | | | | | |

**eTable 2.** **Spearman’s Rank Correlations for biomarker fold change between 72 and 24 hours post-arrest and cognitive instruments**

| **Instruments** | **p-tau^181^** | | **Aβ40** | | **Aβ42** | | **NfL** | | **t-tau** | |
| --- | --- | --- | --- | --- | --- | --- | --- | --- | --- | --- |
|  | rho | Degree of correlation | rho | Degree of correlation | rho | Degree of correlation | rho | Degree of correlation | rho | Degree of correlation |
| MMSE | 0.13 | Small | -0.03 | Trivial | -0.10 | Small | -0.09 | Trivial | -0.04 | Trivial |
| RBMT | 0.04 | Trivial | -0.12 | Small | -0.10 | Small | -0.13 | Small | -0.15* | Small |
| SDMT | 0.13 | Small | -0.03 | Trivial | -0.07 | Trivial | -0.02 | Trivial | -0.04 | Trivial |
| FAB | -0.02 | Trivial | 0.04 | Trivial | -0.06 | Trivial | -0.13 | Small | -0.21** | Small |
| * p < 0.05; ** p < 0.01; *** p < 0.001; Abbreviations: p-tau^181^ – phosphorylated tau at threonine 181, Aβ40 – Amyloid-β 40, Aβ42 – Amyloid-β 42, NfL – neurofilament light chain, t-tau – total tau, MMSE – Mini-Mental State Examination, RBMT – Rivermead Behavioural Memory Test, SDMT – Symbol Digit Modalities Test, FAB – Frontal Assessment Battery. | | | | | | | | | | |
